# Perceived stigma, emotional resilience, and depressive/anxiety symptoms across school stages: a cross-sectional study

**DOI:** 10.3389/fpsyt.2026.1726658

**Published:** 2026-02-11

**Authors:** Shu-Ping Fang, Jian-Jun Luo, Cong Wang, Yi-Hao Liu, Yi-Yue Yang, Lie Zhou, Hui Jin, Yun Xiao, Yang Wen, Jawad Ahmad, Wei Wang, Jia Cai, Qian-Qian Tian, Guo-Qing Jiang, Mao-Sheng Ran

**Affiliations:** 1 Mental Health Center, West China Hospital, Sichuan University, Chengdu, Sichuan, China; 2 Department of Social Psychiatry, West China Hospital, Sichuan University, Chengdu, Sichuan, China; 3 Chongqing Mental Health Center, Chongqing, China; 4 Chengdu Fourth People’s Hospital, Chengdu, Sichuan, China; 5 Department of Social Work and Social Administration, The University of Hong Kong, Hong Kong, Hong Kong SAR, China

**Keywords:** adolescents, anxiety, depression, emotional resilience, stigma, stress

## Abstract

**Introduction:**

Adolescent psychological distress rises sharply. Stigma may inflict harms that exceed the disorders themselves; stress and emotional resilience is pivotal for mental health. Yet how perceived stigma, stress, resilience, and symptoms interact across school stages remains underexplored. We therefore examined stage-specific prevalence and interrelations of perceived stigma, perceived stress, emotional resilience, depression, and anxiety.

**Methods:**

We conducted a cross-sectional, school-based online survey from November 2024 to February 2025 among students in Chongqing, Sichuan, and Hubei. All students in participating schools were invited without sex or age restrictions. The scales included the Perceived Devaluation and Discrimination (PDD), the Stress Numerical Rating Scale–11 (SNRS-11), the Adolescents’ Emotional Resilience Questionnaire (AERQ), the Patient Health Questionnaire-9 (PHQ-9) and the Generalized Anxiety Disorder-7(GAD-7). Analyses comprised descriptive statistics (for stigma, stress, resilience, and mental-health), Pearson and partial correlations (age/sex-adjusted), and mediation modeling.

**Results:**

A total of 86,513 students were included. Clear stage gradients emerged: junior high showed the highest prevalence of PHQ-9/GAD-7 ≥10 and greater stigma and stress, whereas university showed the lowest levels; girls, especially in junior high, bore the greatest burden. In mediation models, stigma was associated with lower resilience, and lower resilience was linked to higher depressive and anxiety symptoms; indirect pathways were significant for both outcomes. The direct effect of stigma was negative overall, with stage-dependent variations: positive in junior/senior high and negative in university, suggesting heterogeneous effects by school stage.

**Conclusion:**

Findings highlight emotional resilience as a principal pathway linking stigma to depressive and anxiety symptoms, with stronger mediation at later school stages. This supports a dual-track approach to school mental health: anti-stigma and help-seeking initiatives alongside resilience-enhancement embedded in curricula and school climate. Limitations include the cross-sectional design, reliance on self-report, potential selection/nonresponse bias from voluntary online participation, and under-representation of out-of-school youth. Prospective longitudinal or intervention studies with causal-mediation frameworks and broader sampling are warranted to clarify mechanisms across developmental stages.

## 1 Introduction

Psychological distress is a state of emotional suffering typically characterized by symptoms of depression and anxiety, while also encompassing broader emotional and behavioral difficulties (1). Adolescence is a developmental period marked by steeply rising incidence and peak onset of multiple psychiatric disorders, including major depressive disorder (MDD) and anxiety disorder (2). MDD is a leading contributor to disease burden and disability among young people worldwide, with over 40% of first episodes occurring before age 20 (3). Population estimates underscore this early burden: the lifetime prevalence of depressive disorder is approximately 12% among U.S. adolescents aged 13–18 and 15% at age 19 in the Netherlands (4, 5). Globally, the adolescent prevalence of anxiety and depressive disorders is broadly comparable (6). Although psychological distress is prevalent, it is compounded by the pervasive burden of stigma (7), Current evidence indicates that social stigma is positively associated with depressive symptomatology, with higher stigma linked to greater depression severity (8).

Stigma associated with mental disorders is a socially constructed phenomenon that marginalizes individuals perceived to deviate from normative behavior (7). A Lancet report suggests that the harms of stigma may, at times, surpass those of the mental disorders themselves (9). As a result, Mental-health–related stigma remains a complex and pervasive global challenge (10).

Stress, triggered by an imbalance in homeostasis or external adverse challenges (11), is widely regarded as a key precipitant of mood disorders and mental health-related stigma (10, 11). The capacity to adapt to stress—emotional resilience—a key factor in the maintenance of mental health (12). This variability in stress responsiveness may account for the considerable heterogeneity in individual reactions, where certain individuals exhibit minimal negative consequences following exposure to stress, while others experience a pronounced exacerbation of detrimental effects (13). Empirical work identifies academic stage, and depression emerged as significant factors for mental health-related stigma (10). Moreover, depressive symptoms and perceived devaluation appear to be bidirectionally related, suggesting that affective state may shape stigma experiences and vice versa (14), while ego-resilience mediates the association between stigma and depressive symptoms (8). Additionally, Several studies of adolescent depression stigma have found that boys endorse higher levels of personal depression stigma (15), which may partly account for the observed sex differences in the prevalence of mood disorders.

Individuals with mental health conditions are frequently subjected to stigma and discrimination in their local communities worldwide (16), and cultures with high collectivism may be associated with elevated levels of internalized stigma (17), thereby intensifying the barriers to public expression and help-seeking (7). In Chinese culture, deeply rooted in the concept of saving “face” (18), individuals with mental health conditions are frequently reluctant to pursue face-to-face psychological treatment (19).

Because stigma is a principal deterrent to students’ help-seeking (20) and a major barrier to addressing mental-health problems in school settings (21), the contemporary literature has largely concentrated on the help-seeking domain (22–31). Yet important gaps persist. Most research on stigma has primarily concentrated on schizophrenia and psychosis-related disorders, while mood disorders such as depression and anxiety have received considerably less attention (32). Although understanding the stigma associated with mental illnesses in adolescents is essential, studies focusing on stigma within this population remain limited (33). Finally, stigma related to mental illnesses may be age-sensitive, but any such effects are likely obscured by the narrow adolescent age ranges commonly sampled; this underscores the need to investigate stigma across a broader developmental span (6).

Accordingly, our study aimed to: (1) estimate, across sex and school stage, the prevalence of perceived stigma, perceived stress, emotional resilience, and mental health status (depression and anxiety; (2) analyze correlations among perceived stigma, stress, emotional resilience, and mental health (depression and anxiety); and (3) test a mediation pathway to determine whether perceived stress/stigma mediates the association between perceived stigma and mental health.

## 2 Materials and methods

### 2.1 Study design and participants

This study is a population-based cross-sectional investigation involving adolescents and young adults from middle schools, high schools, colleges and universities across the provinces of Chongqing, Sichuan, and Hubei. To ensure the rigor of the research, invitation letters were sent to schools in these provinces before data collection began. Participation in the study was voluntary, and ultimately, 180 schools consented to take part. All students studying at these schools, regardless of gender, age, or ethnicity, were invited to participate in the survey. Data collection occurred between November 2024 and February 2025, with data being gathered through an online survey platform. The online self-assessment questionnaire was initially distributed by Wenjuanxing via email to teachers or professors within the participating institutions. These educators then disseminated the survey link to their students, inviting them to voluntarily participate in the study. Informed consent was obtained from all participants prior to completing the online self-report questionnaire. Students who chose not to participate were able to decline on the first page of the survey, at which point the survey was automatically terminated. Throughout the questionnaire completion process, teachers provided no assistance or incentives, allowing students to complete the survey independently. The study complied with the Declaration of Helsinki and was approved by the Medical Ethics Committee of West China Hospital, Sichuan University.

### 2.2 Sociodemographic characteristics

Sociodemographic information was collected using a study-specific questionnaire covering both participant and family characteristics. Variables included age, sex, ethnicity, grade level, academic major (for university students), region of residence, marital/romantic relationships, parents’ marital status, parental employment status, single-child or not, economic status, personal medical history, and family history of psychotic disorder. All data were primarily self-reported by the students.

### 2.3 Perceived stigma

The Perceived Devaluation and Discrimination (PDD) scale measures individuals’ perceptions of how “most people” view and would treat persons with mental illness (34). In this study, the PDD included 13 items rated on a 4-point scale (1 = “totally agree” to 4 = “totally disagree”), with higher total scores indicating greater perceived stigma. In the present sample, the scale demonstrated acceptable internal consistency (Cronbach’s α = 0.702).

### 2.4 Perceived stress

The Stress Numerical Rating Scale–11 (SNRS-11) is a single-item measure of perceived stress rated on an 11-point scale from 0 (“no stress”) to 10 (“the worst stress imaginable”) (35). Higher scores indicate greater perceived stress. The SNRS-11 is brief, easy to administer, and well-suited to surveys and clinical settings that require a rapid, quantitative index of stress.

### 2.5 Emotional resilience

The Adolescents’ Emotional Resilience Questionnaire (AERQ) captures two core domains of resilience—positive emotion generation and recovery from negative affect—across 11 items (36). Responses are recorded on a 6-point Likert scale (1 = strongly disagree/completely inconsistent to 6 = strongly agree/completely consistent), with higher scores indicating greater resilience. Item scores are summed to yield a total score of 11–66. Prior studies have reported strong psychometric performance for the AERQ (37), and in the current sample it demonstrated good internal consistency (Cronbach’s α = 0.818), supporting its validity as a measure of adolescent emotional resilience.

### 2.6 Depression

The Patient Health Questionnaire-9 (PHQ-9) is a 9-item self-report measure of depressive symptoms over the past two weeks (38). Items are scored 0–3 (total 0–27), with higher scores indicating greater severity. Common cutoffs are 5, 10, 15, and 20 for mild, moderate, moderately severe, and severe depression; a score ≥10 often indicates probable major depression. In our sample, the PHQ-9 demonstrated excellent internal consistency (Cronbach’s α = 0.936).

### 2.7 Anxiety

The Generalized Anxiety Disorder-7 (GAD-7) is a 7-item self-report questionnaire designed to assess the severity of anxiety symptoms over the past two weeks (39). Each item is scored on a 4-point scale (0 = “not at all” to 3 = “nearly every day”), yielding a total score ranging from 0 to 21. Higher scores indicate greater anxiety severity, with cutoffs of 5, 10, and 15 representing mild, moderate, and severe anxiety, respectively. In the present study, the GAD-7 demonstrated excellent internal consistency (Cronbach’s α = 0.958).

### 2.8 Statistical analysis

All analyses were conducted using SPSS version 29. Continuous variables with normal distributions are reported as mean ± standard deviation (SD) and compared using one-way ANOVA; non-normally distributed continuous variables are presented as median (interquartile range, IQR). Categorical variables are summarized as counts and percentages and compared using Pearson’s chi-square test. Mediation analyses were conducted using the PROCESS macro (version 5). Stigma was specified as the independent variable (X), psychological resilience as the mediator (M), and PHQ-9 or GAD-7 scores as the outcomes (Y). To examine whether there are differences across school stages, we conducted separate mediation analyses for each stage. We report unstandardized coefficients and R². The bootstrap method produces 95% bias-corrected CI for these effects from 5000 re-sample of the data.

## 3 Results

### 3.1 General information

A total of 94,787 students were invited to participate in the survey, with 6,611 opting not to participate. Following a thorough review and the exclusion of invalid responses from the 88,176 completed surveys, data from 86,513 students were ultimately included in the analysis (junior high group: 13,100; senior high group: 23,058; university group: 50,355). The overall age was 17.20 ± 2.299 years; group means were 13.26 ± 1.023, 16.00 ± 0.924, and 18.79 ± 1.085 years for junior high, senior high, and university. Females accounted for 59.0% of the sample (*p* < 0.001). Most participants were of Han ethnicity (71.4%) and held a rural registered residence (74.6%), with significant stage-wise heterogeneity (both *p* < 0.001). Overall grade distribution was: first year 51.2%, second year 35.2%, third year 13.2%, and fourth/fifth year 0.4% (*p* < 0.001). Among university students, 33.4% majored in medical specialties and 66.6% in non-medical specialties. The majority were not in a romantic relationship (85.2%) and were unmarried (99.6%) (*p* < 0.001). Parents were married in 80.6% of cases (*p* < 0.001). Monthly household income most commonly fell in 2041–4999 RMB (37.9%) and 5000–9999 RMB (28.3%), differing significantly across school stages (*p* < 0.001). Fathers and mothers were employed in 68.4% and 59.0% of families, respectively (father: *p* < 0.001; mother: *p* = 0.069). A lifetime clinician diagnosis of psychotic disorder was reported by 4.4%. Family history of psychosis was positive in 1.7%, negative in 85.4%, and unclear in 12.9%, with significant differences by stage (*p* < 0.001). Detailed participant characteristics are presented in **Table 1**, and descriptive statistics for the scale scores are reported in **Supplementary Table S1**.

**Table 1:** Demographic and relevant characteristics of the participants.

Variable	Overall	Junior high	Senior high	University	*P* value
Sample size (n)	86513	13100	23058	50355	
Age, Mean ± SD, year	17.20 ± 2.299	13.26 ± 1.023	16.00 ± 0.924	18.79 ± 1.085	<0.001
Sex					<0.001
Male (%)	35462 (41.0%)	6764 (51.6%)	10208 (44.3%)	18490 (36.7%)	
Female (%)	51051 (59.0%)	6336 (48.4%)	12850 (55.7%)	31865 (63.3%)	
Ethnic group					<0.001
Han (%)	61792 (71.4%)	3652 (27.9%)	13829 (60.0%)	44311 (88.0%)	
Other (%)	24721 (28.6%)	9448 (72.1%)	9229 (40.0%)	6044 (12.0%)	
Registered residence					<0.001
Rural (%)	64574 (74.6%)	9462 (72.2%)	18377 (79.7%)	36735 (73.0%)	
Urban (%)	21939 (25.4%)	3638 (27.8)	4681 (20.3%)	13620 (27.0%)	
Grade					<0.001
First year (%)	44310 (51.2%)	4330 (33.1%)	9930 (43.1%)	30050 (59.7%)	
Second year (%)	30444 (35.2%)	4497 (34.3%)	9898 (42.9%)	16049 (31.9%)	
Third year (%)	11405 (13.2%)	4273 (32.6%)	3230 (14.0%)	3902 (7.7%)	
Fourth & Fifth year (%)	354 (0.4%)	–	–	354 (0.7%)	
Major					–
Medical specialty (%)	–	–	–	16838 (33.4%)	
Non-medical specialty (%)	–	–	–	33517 (66.6%)	
Marital/romantic relationships					
Not in a romantic relationship (%)	73677 (85.2%)	12773 (97.5%)	21967 (95.3%)	38937 (77.3%)	<0.001
Not married (%)	86156 (99.6%)	13100 (100%)	23058 (100%)	49998 (99.3%)	–
Marital status of parents					<0.001
Married (%)	69707 (80.6%)	11128 (84.9%)	18271 (79.2%)	40308 (80.0%)	
Other (%)	16806 (19.4%)	1972 (15.1%)	4787 (20.8%)	10047 (20.0%)	
Family Income (monthly)					<0.001
≤2040 RMB (%)	16284 (18.8%)	2676 (20.4%)	3938 (17.1%)	9670 (19.2%)	
2041–4999 RMB (%)	32778 (37.9%)	4662 (35.6%)	9515 (41.3%)	18601 (36.9%)	
5000–9999 RMB (%)	24511 (28.3%)	3911 (29.9%)	6661 (28.9%)	13939 (27.7%)	
10000–19999 RMB (%)	9376 (10.8%)	1381 (10.5%)	2162 (9.4%)	5833 (11.6%)	
20000–39999 RMB (%)	2290 (2.6%)	306 (2.3%)	514 (2.2%)	1470 (2.9%)	
>40,000 RMB (%)	1274 (1.5%)	164 (1.3%)	268 (1.2%)	842 (1.7%)	
Single-child					<0.001
Yes (%)	17290 (20.0%)	1932 (14.7%)	3666 (15.9%)	11692 (23.2%)	
No (%)	69223 (80.0%)	11168 (85.3%)	19392 (84.1%)	38663 (76.8%)	
Father’s employment status					<0.001
In employment (%)	59150 (68.4%)	9249 (70.6%)	15727 (68.2%)	34174 (67.9%)	
Other (%)	27363 (31.6%)	3851 (29.4%)	7331 (31.8%)	16181 (32.1%)	
Mother’s employment status					0.069
In employment (%)	51066 (59.0%)	7794 (59.5%)	13714 (59.5%)	29558 (58.7%)	
Other (%)	35447 (41.0%)	5306 (40.5%)	9344 (40.5%)	20797 (41.3%)	
History of psychotic disorder					<0.001
Diagnosed (%)	3781 (4.4%)	637 (4.9%)	1286 (5.8%)	1858 (3.7%)	
Other (%)	82732 (95.6%)	12463 (95.1%)	21772 (94.2%)	48497 (96.3%)	
Family history of psychosis					<0.001
Positive (%)	1453 (1.7%)	213 (1.6%)	391 (1.7%)	849 (1.7%)	
Negative (%)	73867 (85.4%)	10255 (78.3%)	18793 (81.5%)	44819 (89.0%)	
Unclear (%)	11193 (12.9%)	2632 (20.1%)	3874 (16.8%)	4687 (9.3%)	

### 3.2 Stage-specific profiles of stigma, stress, resilience, and depression/anxiety

We defined clinically meaningful depression/anxiety as PHQ-9/GAD-7 ≥10 (39, 40). Because PDD, SNRS-11, and AERQ lack validated cut-offs, we summarized their distributions using quartiles (descriptive only, not severity). For each school stage, we report the number and within-stage percentage of participants falling in each quartile, with sex-stratified tabulations.

The proportion of students with PDD ≥35 was significantly higher in junior high (20.6%, n = 2,693) than in senior high (16.4%, n = 3,787) and university (16.9%, n = 8,515) (**Supplementary Table S2a**); this pattern persisted after sex stratification (**Supplementary Table S2b**). The distribution of the stress (SNRS-11) score is similar: junior high students were overrepresented at higher score bands, whereas university students were concentrated in the lower bands (**Supplementary Table S3a**). In sex- and stage-stratified analyses, females and junior high students were more likely to fall in the highest stress quartile (Q4), whereas males and university students exhibited the lowest stress burden (**Supplementary Table S3b**). In addition, resilience (AERQ) scores were comparatively higher in the junior high group (**Supplementary Tables S4a, b**).

Significant between-stage and sex differences were observed in the distributions of GAD-7 and PHQ-9 categories. In the overall sample, 90.2% (n =78,011) had GAD-7 <10, whereas 9.8% (n =8,502) had GAD-7 ≥10. The prevalence of GAD-7 ≥10 was highest in the junior-high group (15.2%, n =1,988), followed by senior-high (13.8%, n =3,179), and lowest in the university group (6.6%, n =3,335). Among females, the prevalence of GAD-7 ≥10 was 19.4% in junior high, declining to 14.8% in senior high and 6.3% in university. For depression, the proportion with PHQ-9 ≥10 was also lowest in the university group (10.5%, n =5,288), significantly below the junior- and senior-high groups. In sex-stratified analyses, junior-high girls had the highest prevalence of PHQ-9 ≥10 (26.2%, n =1,661), whereas university girls had the lowest (10.0%, n =3,192). Detailed counts and percentages are provided in **Tables 2a**, **b**.

**Table 2a:** Distribution of GAD-7/PHQ-9 scores across school stages.

Variable	Overall (n=86513)	Junior high (n=13100)	Senior high (n=23058)	University (n=50355)	X^2^	*P* value	*Post hoc* analysis
GAD-7					1414.262	<0.001	
GAD-7<10 (%)	78011 (90.2%)	11112 (84.8%)	19879 (86.2%)	47020 (93.4%)			a,b,c
GAD-7≥10 (%)	8502 (9.8%)	1988 (15.2%)	3179 (13.8%)	3335 (6.6%)			a,b,c
PHQ-9					1673.420	<0.001	
PHQ-9<10 (%)	73823 (85.3%)	10371 (79.2%)	18385 (79.7%)	45067 (89.5%)			b,c
PHQ-9≥10 (%)	12690 (14.7%)	2729 (20.8%)	4673 (20.3%)	5288 (10.5%)			b,c

a, significant difference between the junior high group and the senior high group; b, significant difference between the junior high group and the university group; c, significant difference between the senior high group and the university group.

**Table 2b:** Sex-stratified distribution of GAD-7/PHQ-9 scores across school stages.

Variable		Overall (n=86513)	Junior high (n=13100)	Senior high (n=23058)	University (n=50355)	X^2^	*P* value	*Post hoc* analysis
Male (%)						250.270	<0.001	
	GAD-7<10	32111 (90.6%)	6003 (88.7%)	8936 (87.5%)	17172 (92.9%)			b,c
	GAD-7≥10	3351 (9.4%)	761 (11.3%)	1272 (12.5%)	1318 (7.1%)			b,c
Female (%)						1417.177	<0.001	
	GAD-7<10	45900 (89.9%)	5109 (80.6%)	10943 (85.2%)	29848 (93.7%)			a,b,c
	GAD-7≥10	5151 (10.1%)	1227 (19.4%)	1907 (14.8%)	2017 (6.3%)			a,b,c
Male (%)						312.083	<0.001	
	PHQ-9<10	30380 (85.7%)	5696 (84.2%)	8290 (81.2%)	16394 (88.7%)			a,b,c
	PHQ-9≥10	5082 (14.3%)	1068 (15.8%)	1918 (18.8%)	2096 (11.3%)			a,b,c
Female (%)						1672.073	<0.001	
	PHQ-9<10	43443 (85.1%)	4675 (73.8%)	10095 (78.6%)	28673 (90.0%)			a,b,c
	PHQ-9≥10	7608 (14.9%)	1661 (26.2%)	2755 (21.4%)	3192 (10.0%)			a,b,c

a, significant difference between the junior high group and the senior high group; b, significant difference between the junior high group and the university group; c, significant difference between the senior high group and the university group.

### 3.3 Pearson correlations among stigma, stress, resilience, and depression/anxiety

Correlation analyses showed that the association between PDD and SNRS-11 was statistically significant but weak (r = 0.068, *p* < 0.001). Similarly, the correlations of PDD with PHQ-9 and GAD-7 were weak (r = 0.084, *p* < 0.001 and r = 0.080, *p* < 0.001, respectively).

But AERQ showed stronger associations overall: it was inversely correlated with PDD (r = −0.269) and more strongly with PHQ-9 and GAD-7 (both r = −0.427; all *p* < 0.001), indicating that higher resilience relates to lower perceived stigma and fewer depressive/anxiety symptoms (N = 86,513). We then conducted partial correlation analyses, adjusting only for age and sex because additional covariates had negligible influence. Results were essentially unchanged: PDD–resilience partial r = −0.223 (*p* < 0.001); PHQ-9 partial r = −0.424 (*p* < 0.001); GAD-7 partial r = −0.426 (*p* < 0.001). The PDD–AERQ link was only modestly attenuated (−0.269→−0.223), whereas associations with symptoms remained stable. Detailed results are provided in **Table 3**.

**Table 3:** Partial correlations among PDD, resilience, PHQ-9, and GAD-7 (N = 86,513).

Variable	PDD	AERQ	PHQ-9	GAD-7
PDD	1	−0.223*	0.077*	0.082*
AERQ		1	−0.424*	−0.426*
PHQ-9			1	0.878*
GAD-7				1

*p<0.001

### 3.4 The mediating role of emotional resilience in the relationship between stigma and mental health

**Figure 1:**
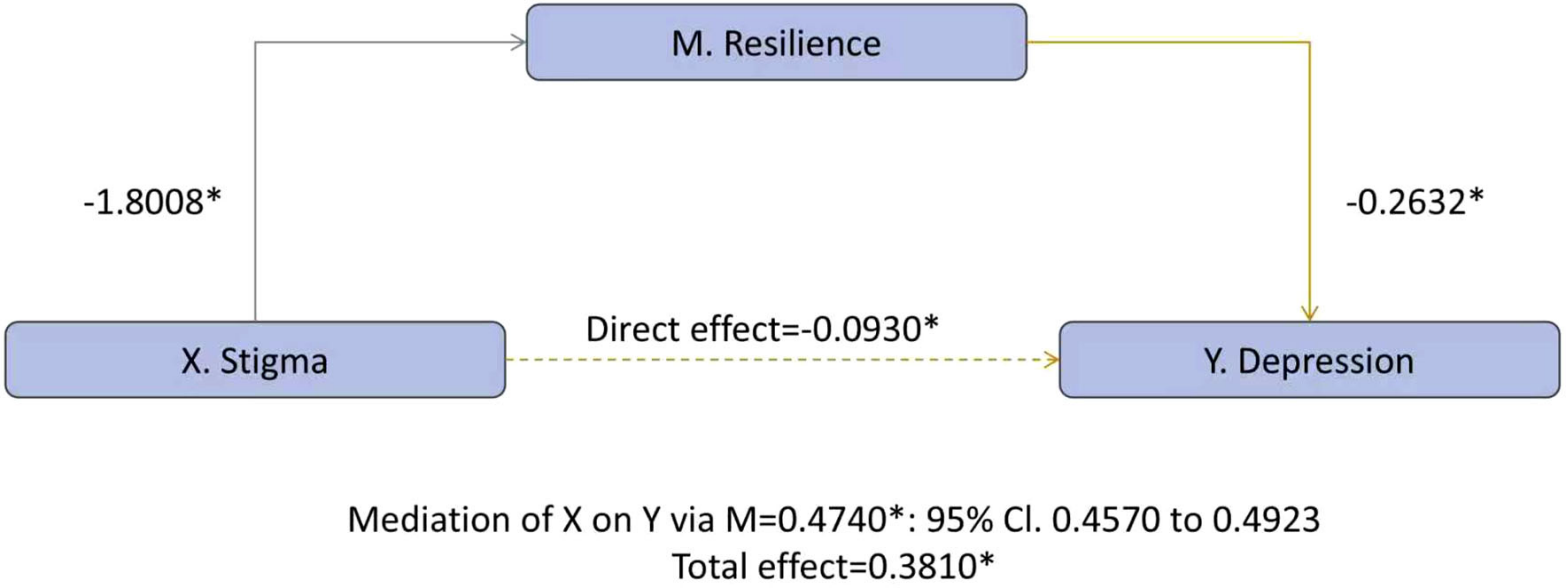
Resilience mediates the association between stigma and depression *p<0.001.

Using the SPSS mediation test procedure, we tested whether emotional resilience mediates the association between perceived stigma and mental health. Perceived stigma negatively predicted emotional resilience (a = −1.8008, *p* < 0.001). Resilience, in turn, was inversely associated with depression (b = −0.2632, *p* < 0.001). The indirect effect was significant (ab = 0.4740, bias-corrected 95% CI 0.4570–0.4923), while the direct effect became negative (c′ = −0.0930, *p* < 0.001) despite a positive total effect (c = 0.3810, *p* < 0.001), indicating inconsistent mediation(suppression). Explained variance increased from R² = 0.0093 (total-effect model) to R² = 0.1831 when resilience was included, suggesting that reduced resilience is the important pathway through which stigma relates to depressive symptom (**Figure 1**).

**Figure 2:**
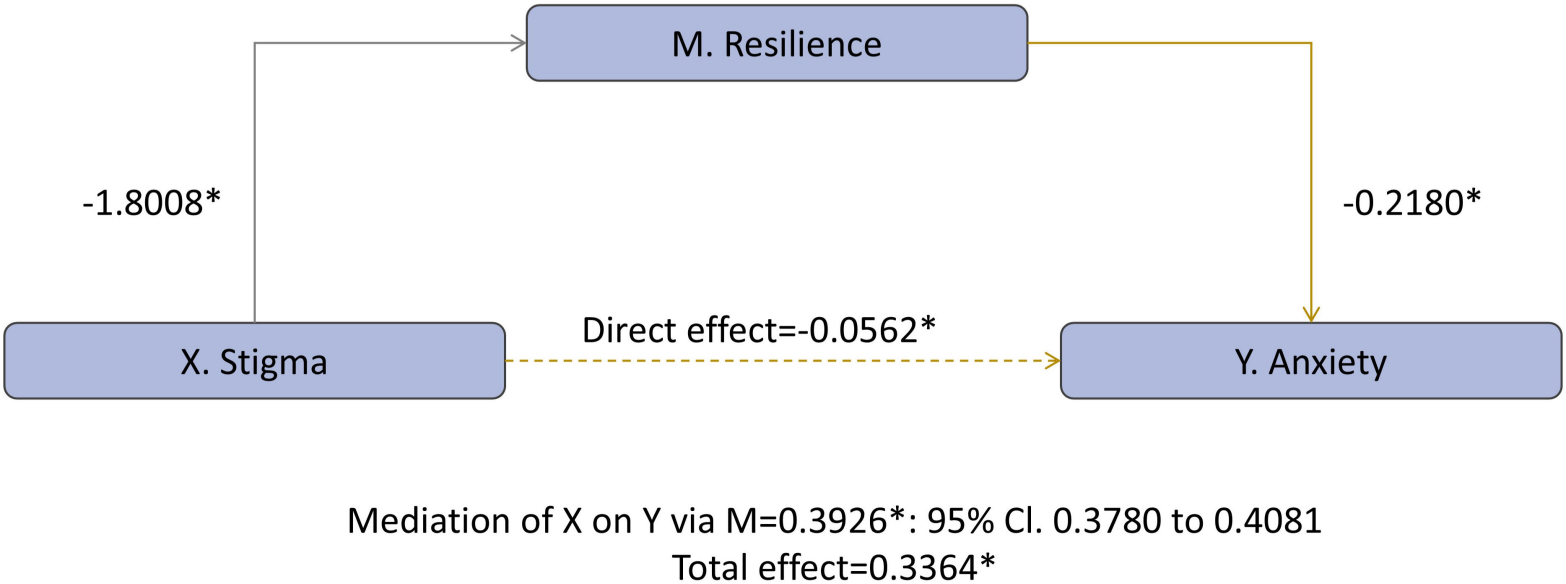
Resilience mediates the association between stigma and anxiety *p<0.001.

Parallel findings emerged for anxiety (**Figure 2**): resilience related inversely to anxiety (b = −0.2180, *p* < 0.001), yielding a significant indirect effect (ab = 0.3926, bias-corrected 95% CI 0.3780–0.4081), a negative direct effect (c′ = −0.0562, *p* < 0.001), and a positive total effect (c = 0.3364, *p* < 0.001), again evidencing inconsistent mediation (suppression). With resilience included, R² increased from 0.0093 to 0.1833, underscoring reduced resilience as an important pathway linking stigma to anxiety as well.

**Figure 3:**
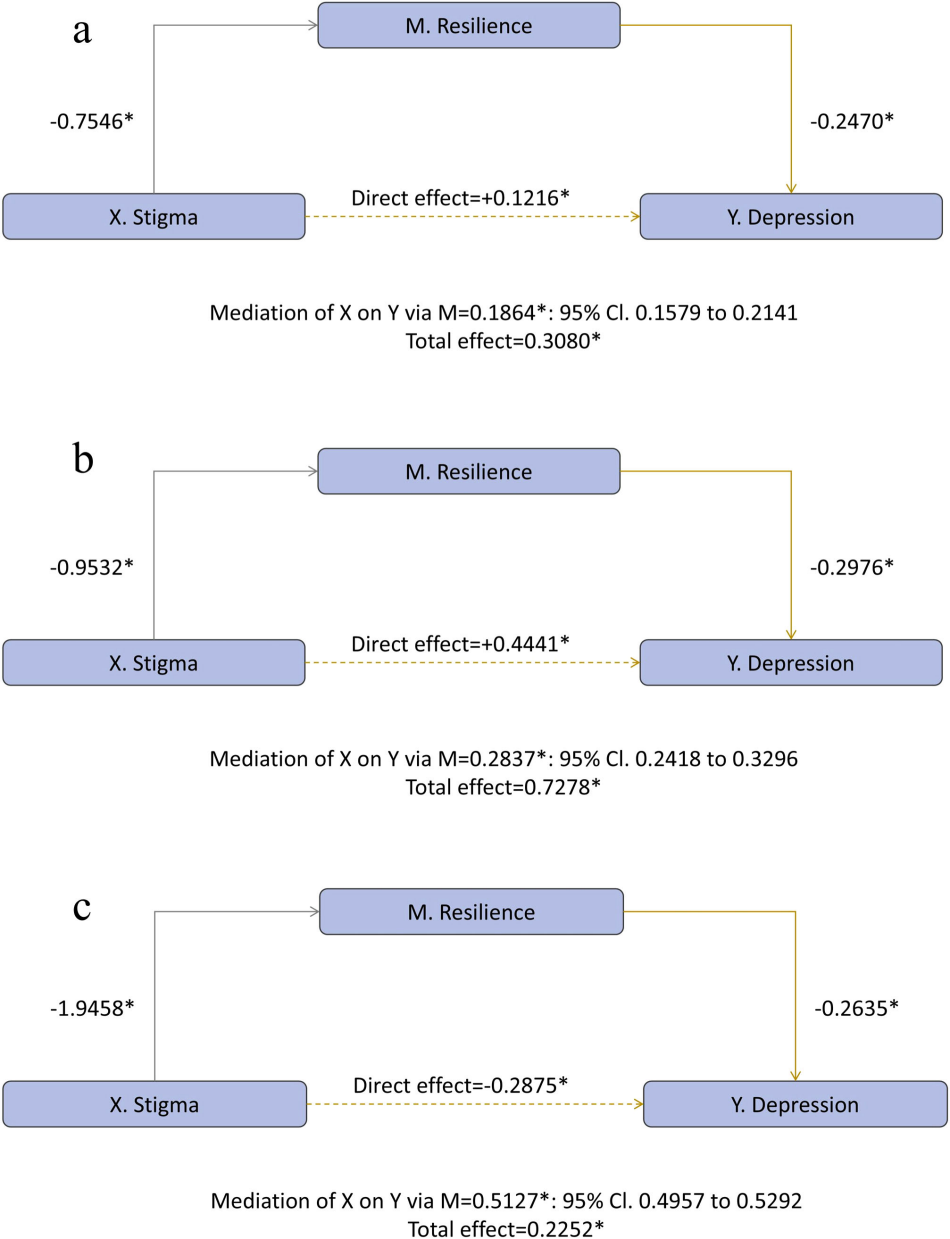
School-stage-specific moderated mediation of stigma on depression through emotional resilience. **(a)** Junior high; **(b)** Senior high; **(c)** University.

Next, to examine differences across school stages, we conducted separate mediation analyses for each stage. In the junior-high group (**Figure 3a**), stigma predicted lower resilience (a = −0.7546) and resilience was inversely related to depression (b = −0.2470), yielding a significant indirect effect (ab = 0.1864, 95% CI 0.1579–0.2141) alongside a positive direct effect (c*′* = 0.1216) and total effect (c = 0.3080; all *p* <.001). In the senior-high group (**Figure 3b**), both paths strengthened (a = −0.9532; b = −0.2976), increasing the indirect effect (ab = 0.2837, 95% CI 0.2418–0.3296) while the direct effect remained positive (c′ = 0.4441; total c = 0.7278; all *p* < 0.001). In the university group (**Figure 3c**), stigma’s impact on resilience was largest (a = −1.9458) and resilience remained protective (b = −0.2635), producing the strongest indirect effect (ab = 0.5127, 95% CI 0.4957–0.5292); notably, the direct effect reversed (c′ = −0.2875) while the total effect stayed positive (c = 0.2252; all *p* <.001).

**Figure 4:**
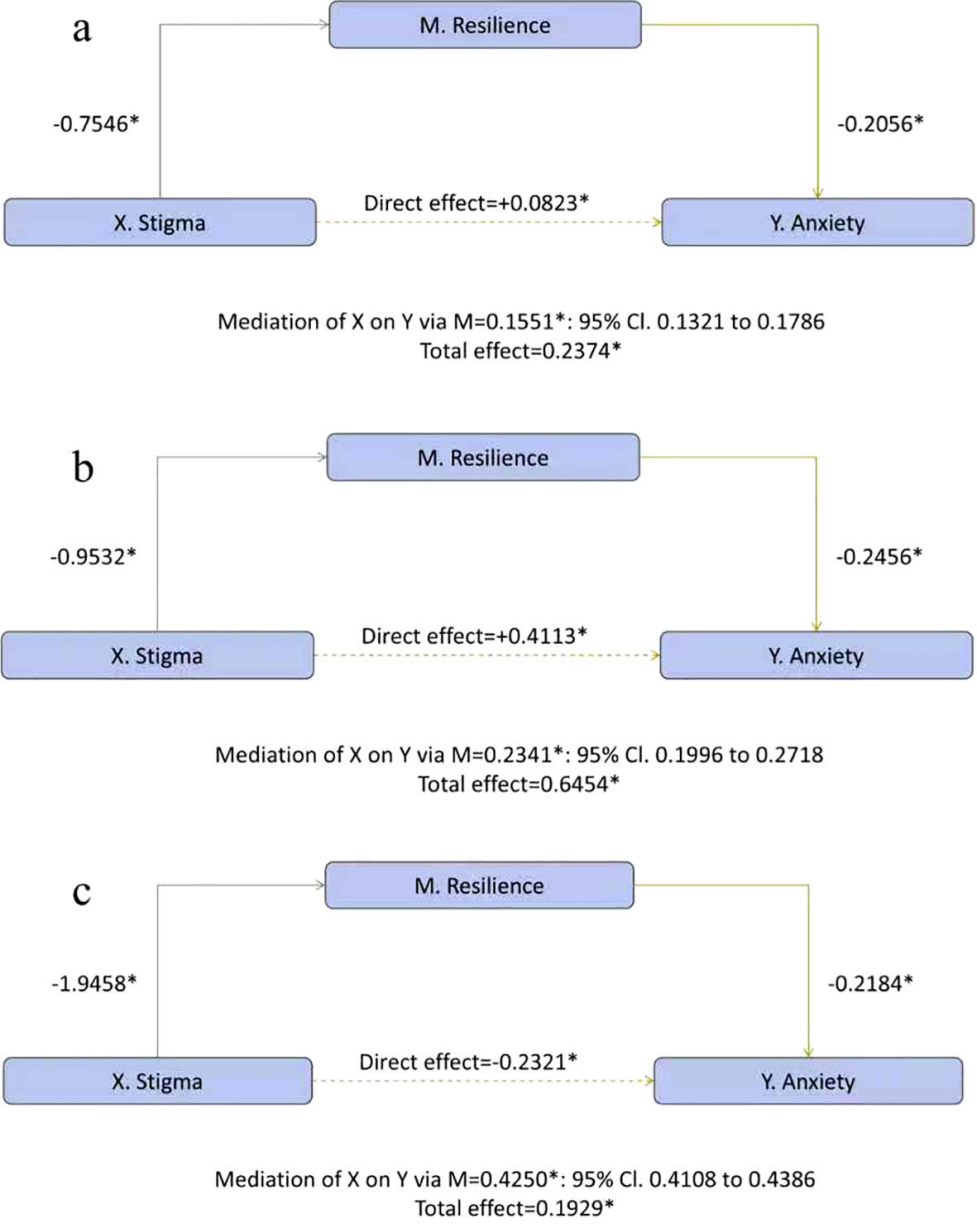
School-stage-specific moderated mediation of stigma on anxiety through emotional resilience. **(a)** Junior high; **(b)** Senior high; **(c)** University.

For anxiety (**Figures 4a-c**), the pattern paralleled the depression model: the a-path (stigma → resilience) was negative and became progressively stronger from junior to senior high to university (−0.7546→−0.9532→−1.9458); the b-path (resilience → anxiety) was uniformly inverse (−0.2056, −0.2456, −0.2184; all *p* < 0.001); and the conditional indirect effect increased across stages (ab=0.1551, 0.2341, 0.4250). The direct path showed the same sign pattern as for depression—positive in junior and senior high (c′=0.0823; 0.4113) but negative in university (c′=−0.2321). However, given that our study is cross-sectional in nature and subject to limitations such as sample selection bias, the interpretation of the results should be made with caution.

## 4 Discussion

In this large-scale cross-sectional survey, a distinct stage gradient emerged: the prevalence of anxiety/depression (GAD-7/PHQ-9 ≥ 10) was highest in junior high school students, who also reported the greatest perceived stress. The prevalence of depression and anxiety, along with higher levels of perceived stress, was notably higher in middle school students compared to university students, supporting the view that stress is a potent precipitant of emotional disorders (41). This pattern plausibly reflects the academic and developmental context of middle school adolescence: students are confronted with highly stressful entrance exams (e.g., high school/university entrance exams) and more parent-supervised living environments. Supporting this notion, a Korean survey of 291,110 students aged 12–18 identified school/career concerns as the most prevalent source of psychological stress; additionally, conflicts with parents and peers and adverse aspects of the family environment further increased adolescents’ vulnerability to mental health problems (42). Our study aligns with previous research, particularly regarding middle school students, where female sex was associated with greater symptom burden (43, 44). Developmentally, adolescence is a period of rapid social, emotional, and cognitive change with key life transitions (45); middle-school students typically fall within this developmental window, and hormonal shifts may partly account for the sex differences in mood disorders observed as early as 12 years and peaking around 17 (46).

Although perceived stress was significantly correlated with mental health, its association with perceived stigma was only weak, whereas emotional resilience showed a much stronger linkage with both perceived stigma and mental health. During adolescence, the influence of peers and peer norms increases dramatically (47); in the school environment, both physical and relational bullying can impact mental health through the process of stigmatization (48). In contrast to some earlier studies, we observed higher perceived stigma among younger students (15). This may be due to individuals with lower educational backgrounds being more likely to have limited understanding of mental health conditions, and consequently, have minimal knowledge about the causes, treatment, and recovery of mental illnesses, leading to higher levels of self-stigma (49, 50). Although awareness of mental illness is improving, stigma remains deeply entrenched (50). Perceived stigma may have a detrimental impact on the lives of adolescents with emotional issues, such as damaging their interactions with others, leading to social isolation, feelings of inferiority, or a diminished sense of self-worth, which could, in turn, amplify the negative characteristics of emotional disorders (51–53). In our mediation models, the overall direct effect of perceived stigma on mental symptoms (X→Y) was negative, diverging from prior literature. However, after moderating by school stage, direct effects were positive in junior and senior high but negative in university. This pattern suggests that, among middle/high school students, our findings align with prior work showing a positive association between stigma and mental health problems (8), whereas the sign reversal in university suggests a different interplay during the transition to adulthood—potentially indicated that the stigma–mental health relationship is not uniform across development, with the mediating role of resilience differing by school stage. This may be because most middle school students are minors who require parental supervision, and they are more likely to seek help from their parents regarding psychological issues rather than from professional healthcare providers (54). In contrast, with mental health concerns among college students increasingly garnering societal attention, most universities have established psychological counseling services to meet students’ psychological health needs (55). Additionally, college students generally experience less stress and may possess more psychological knowledge and social support. Perceived stigma may, to some extent, motivate college students to adopt coping mechanisms to alleviate psychological pressure. The relationship between stigma and mental health is not uniform across different developmental stages, and the mediating role of psychological resilience varies depending on the school stage. Therefore, during the transition to adulthood, different interactions emerge, which may reflect changes in biology, cognition, social roles, academic demands, and living environments.

Our data align with previous studies, indicating a negative correlation between stigma and resilience (56), with more severe stigma associated with more severe depressive symptoms (8). Additionally, stigma was positively correlated with lower treatment adherence (57). Evaluating and enhancing resilience may be beneficial for medication adherence (58), as higher resilience can serve as a protective factor against emotional disorders (59). Although the specific mechanisms through which perceived stigma and psychological resilience affect emotional outcomes remain unclear, early intervention for adolescents with emotional issues is essential (60). This is particularly significant given that a reduction in stigmatizing beliefs over time has been associated with improvements in both functioning and quality of life (61). Active interventions may represent a promising strategy for enhancing mental health literacy, reducing stigmatizing attitudes and social distance toward individuals with mental health issues (62). A recent meta-analysis indicates that anti-stigma interventions targeting youth can produce short-term benefits (63), while interventions designed to enhance resilience can lead to improvements in mental health to a certain degree (37). The integration of stigma-focused therapeutic and educational programs with resilience-based interventions is essential for effectively supporting students with emotional symptoms in improving their health outcomes (64).

However, cultural factors must be considered before implementing interventions (52), as stigma varies significantly across geographic and cultural contexts (52), with self-stigma being particularly pronounced in Asian populations (60). This may be related to cultural factors such as the concept of ‘face,’ shame, collectivism, family stigma, and others, which remain powerful determinants of stigma (49, 65). The specific manifestations of stigma in Chinese populations are influenced by cultural beliefs rooted in Confucianism, pejorative etiological beliefs of mental illness, and the prominence of the concept of ‘face’ (49). Furthermore, in collectivist cultures, stigma may be amplified, whereas more individualistic settings may experience reduced stigma intensity (66). Additionally, stigma has been linked to low social capital, diminished relational satisfaction, negative parenting experiences, and familial expressed emotions (61). Therefore, any intervention aimed at reducing stigma and fostering resilience must thoroughly consider cultural differences and tailor interventions to address the evolving social landscape and the unique needs of individuals (66).

This study has limitations. Its cross-sectional design precludes establishing temporal precedence; accordingly, the reported “indirect effects” should be interpreted as statistical rather than causal mediation. All outcomes were derived from self-report instruments, for PDD, SNRS-11, and AERQ there are no unified clinical cut-offs, so quartiles were used purely descriptively, which may affect interpretability. The sample derived from voluntary, online participation, introducing potential selection and nonresponse bias. Additionally, our school-based approach only partially represents youth, as out-of-school adolescents—who may be numerous (8)—were not captured; importantly, school dropout is associated with higher risks of depression, anxiety, suicidality, lower life satisfaction, substance misuse, poorer general health, higher mortality, and lower socioeconomic status (8). As such, these limitations may affect the accuracy of our conclusions. Finally, the findings of our study may not be generalizable to other ethnic groups or regions, as culture plays a key role in shaping stigma and resilience processes (66).

Future research should employ prospective longitudinal or intervention designs combined with causal mediation approaches; broaden sampling to include out-of-school/offline populations; and pair self-report with clinician-rated or diagnostic assessments, while expanding regional coverage. Additionally, considering cultural and gender factors may enhance the generalizability of the findings. Such work will better elucidate the stigma–resilience–mental health pathway.

## 5 Conclusions

In this large sample of adolescents, perceived stigma was linked to lower emotional resilience, and lower resilience was, in turn, associated with greater depressive and anxiety symptoms. The indirect pathway (stigma → reduced resilience → higher symptoms) strengthened progressively from junior high to university, while the direct effect of stigma on symptoms was positive in junior/senior high but negative in university, indicating stage-dependent inconsistent mediation/suppression. Overall, emotional resilience emerges as a principal pathway connecting stigma to mental-health burden, and its salience appears to increase with educational stage.

These findings suggest that, alongside the growing focus on the impact of stress on mental health, a dual-track approach is necessary for school mental-health promotion. This approach should include implementing anti-stigma and help-seeking initiatives (with particular attention to girls and to junior/senior high students), alongside resilience-enhancement programs embedded in curricula and supported by peers, teachers, and the school climate. Given the cross-sectional design, the observed pathways should be interpreted as indirect associations rather than causal effects. Longitudinal or intervention studies are needed to replicate these patterns across broader populations and to clarify the underlying mechanisms. 

## Data Availability

The raw data supporting the conclusions of this article will be made available by the authors, without undue reservation.
