# Supplementary material for: Perceived stigma, emotional resilience, and depressive/anxiety symptoms across school stages: a cross-sectional study
Source: Front Psychiatry. 2026 Feb 11;17:1726658. doi: 10.3389/fpsyt.2026.1726658 (PMC12947124; doi:10.3389/fpsyt.2026.1726658)
Supplement: Supplementary file 1 [file DataSheet1.docx]

**Supplementary Material**

**Table S1. Descriptive Statistics of Psychometric Scale Scores in the Total Sample**

|  | **Overall** | **Junior high** | **Senior high** | **University** | **F** | ***P* value** | **Post hoc analysis** |
| --- | --- | --- | --- | --- | --- | --- | --- |
|  | 86513 | 13100 | 23058 | 50355 |  |  |  |
| PDD | 32.44(2.606) | 32.57(2.782) | 32.37(2.552) | 32.43(2.583) | 24.453 | <0.001 | a,b,c |
| PHQ-9 | 4.52(5.506) | 5.41(6.368) | 5.66(5.948) | 3.76(4.894) | 1178.214 | <0.001 | a,b,c |
| GAD | 3.40(4.557) | 4.34(5.308) | 4.32(4.948) | 2.73(4.008) | 1342.843 | <0.001 | b,c |
| SNRS-11 | 2.79(2.394) | 3.28(2.633) | 3.12(2.474) | 2.52(2.248) | 834.966 | <0.001 | a,b,c |
| AERQ | 39.85(8.693) | 39.39(9.930) | 38.61(8.590) | 40.54(8.317) | 415.425 | <0.001 | a,b,c |

a: significant difference between the junior high group and the senior high group; b: significant difference between the junior high group and the university group; c: significant difference between the senior high group and the university group.

**Table S2a. Quartile distribution of PDD scores across school stages**

|  | **Overall (n=86513)** | **Junior high (n=13100)** | **Senior high (n=23058)** | **University (n=50355)** | **X^2^** | ***P* value** | **Post hoc analysis** |
| --- | --- | --- | --- | --- | --- | --- | --- |
| PDD scores |  |  |  |  | 174.809 | <0.001 |  |
| ≤31 | 28251(32.7%) | 4285(32.7%) | 7744(33.6%) | 16222(32.2%) |  |  | c |
| 32–33 | 29116(33.7%) | 4145 (31.6%) | 7995(34.7%) | 16976(33.7%) |  |  | a,b,c |
| =34 | 14151(16.4%) | 1977(15.1%) | 3532(15.3%) | 8642(17.2%) |  |  | a,c |
| ≥35 | 14995(17.3%) | 2693(20.6%) | 3787 (16.4%) | 8515(16.9%) |  |  | a,b |

a: significant difference between the junior high group and the senior high group; b: significant difference between the junior high group and the university group; c: significant difference between the senior high group and the university group.

**Table S2b. Sex-stratified quartile distribution of PDD scores across school stages**

|  | **PDD** | **Overall (n=86513)** | **Junior high (n=13100)** | **Senior high (n=23058)** | **University (n=50355)** | **X^2^** | ***P* value** | **Post hoc analysis** |
| --- | --- | --- | --- | --- | --- | --- | --- | --- |
|  |  | 86513 | 13100 | 23058 | 50355 |  |  |  |
| Male |  |  |  |  |  | 112.688 | <0.001 |  |
|  | ≤31 | 10553(29.8%) | 2120(31.3%) | 3155(30.9%) | 5278(28.5%) |  |  | b,c |
|  | 32–33 | 12075(34.1%) | 2158(31.9%) | 3564(34.9%) | 6353(34.4%) |  |  | a,b |
|  | =34 | 6438(18.2%) | 1077(15.9%) | 1747(17.1%) | 3614(19.5%) |  |  | b,c |
|  | ≥35 | 6396(18.0%) | 1409(20.8%) | 1742(17.1%) | 3245(17.6%) |  |  | a,b |
| Female |  |  |  |  |  | 95.793 | <0.001 |  |
|  | ≤31 | 17698(34.7%) | 2165(34.2%) | 4589(35.7%) | 10944(34.3%) |  |  | c |
|  | 32–33 | 17041(33.4%) | 1987(31.4%) | 4431(34.5%) | 10623(33.3%) |  |  | a,b |
|  | =34 | 7713(15.1%) | 900(14.2%) | 1785(13.9%) | 5028(15.8%) |  |  | b,c |
|  | ≥35 | 8599(16.8%) | 1284(20.3%) | 2045(15.9%) | 5270(16.5%) |  |  | a,b |

a: significant difference between the junior high group and the senior high group; b: significant difference between the junior high group and the university group; c: significant difference between the senior high group and the university group.

**Table S3a. Quartile distribution of SNRS-11 scores across school stages**

|  | **Overall (n=86513)** | **Junior high (n=13100)** | **Senior high (n=23058)** | **University (n=50355)** | **X^2^** | ***P* value** | **Post hoc analysis** |
| --- | --- | --- | --- | --- | --- | --- | --- |
| SNRS-11 scores |  |  |  |  | 1439.900 | <0.001 |  |
| ≤1 | 30287(35.0%) | 3933(30.0%) | 7009(30.4%) | 19345(38.4%) |  |  | b,c |
| =2 | 17175(19.9%) | 2392(18.3%) | 4244(18.4%) | 10539(20.9%) |  |  | b,c |
| 3-4 | 19468(22.5%) | 2872(21.9%) | 5406(23.4%) | 11190(22.2%) |  |  | a,c |
| ≥5 | 19583(22.6%) | 3903(29.8%) | 6399(27.8%) | 9281(18.4%) |  |  | a,b,c |

a: significant difference between the junior high group and the senior high group; b: significant difference between the junior high group and the university group; c: significant difference between the senior high group and the university group.

**Table S3b. Sex-stratified quartile distribution of SNRS-11 scores across school stages**

|  | **SNRS-11** | **Overall (n=86513)** | **Junior high (n=13100)** | **Senior high (n=23058)** | **University (n=50355)** | **X^2^** | ***P* value** | **Post hoc analysis** |
| --- | --- | --- | --- | --- | --- | --- | --- | --- |
|  |  | 86513 | 13100 | 23058 | 50355 |  |  |  |
| **Male** |  |  |  |  |  | 356.742 | <0.001 |  |
|  | ≤1 | 14036(39.6%) | 2469(36.5%) | 3595(35.2%) | 7972(43.1%) |  |  | b,c |
|  | =2 | 7169(20.2%) | 1365(20.2%) | 1984(19.4%) | 3820(20.7%) |  |  | c |
|  | 3-4 | 7336(20.7%) | 1390(20.5%) | 2248(22.0%) | 3698(20.0%) |  |  | c |
|  | ≥5 | 6921(19.5%) | 1540(22.8%) | 2381(23.3%) | 3000(16.2%) |  |  | b,c |
| **Female** |  |  |  |  |  | 1465.176 | <0.001 |  |
|  | ≤1 | 16251(31.8%) | 1464(23.1%) | 3414(26.6%) | 11373(35.7%) |  |  | a,b,c |
|  | =2 | 10006(19.6%) | 1027(16.2%) | 2260(17.6%) | 6719(21.1%) |  |  | b,c |
|  | 3-4 | 12132(23.8%) | 1482(23.4%) | 3158(24.6%) | 7492(23.5%) |  |  | - |
|  | ≥5 | 12662(24.8%) | 2363(37.3%) | 4018(31.3%) | 6281(19.7%) |  |  | a,b,c |

a: significant difference between the junior high group and the senior high group; b: significant difference between the junior high group and the university group; c: significant difference between the senior high group and the university group.

**Table S4a. Quartile distribution of AERQ scores across school stages**

|  | **Overall (n=86513)** | **Junior high (n=13100)** | **Senior high (n=23058)** | **University (n=50355)** | **X^2^** | ***P* value** | **Post hoc analysis** |
| --- | --- | --- | --- | --- | --- | --- | --- |
|  | 86513 | 13100 | 23058 | 50355 |  |  |  |
| AERQ scores |  |  |  |  | 1789.510 | <0.001 |  |
| ≤35 | 21633(25.0%) | 2839(21.7%) | 4914(21.3%) | 13880 (27.6%) |  |  | b,c |
| 36-39 | 27001(31.2%) | 3045 (23.2%) | 7108(30.8%) | 16848(33.5%) |  |  | a,b,c |
| 40-43 | 16665(19.3%) | 2506(19.1%) | 4898(21.2%) | 9261(18.4%) |  |  | a,c |
| ≥44 | 21214(24.5%) | 4710(36.0%) | 6138 (26.6%) | 10366(20.6%) |  |  | a,b,c |

a: significant difference between the junior high group and the senior high group; b: significant difference between the junior high group and the university group; c: significant difference between the senior high group and the university group.

**Table S4b. Sex-stratified quartile distribution of AERQ scores across school stages**

|  | **AERQ** | **Overall (n=86513)** | **Junior high (n=13100)** | **Senior high (n=23058)** | **University (n=50355)** | **X^2^** | ***P* value** | **Post hoc analysis** |
| --- | --- | --- | --- | --- | --- | --- | --- | --- |
|  |  | 86513 | 13100 | 23058 | 50355 |  |  |  |
| **Male** |  |  |  |  |  | 861.482 | <0.001 |  |
|  | ≤35 | 8719(24.6%) | 1491(22.0%) | 2165(21.2%) | 5063(27.4%) |  |  | b,c |
|  | 36-39 | 10948(30.9%) | 1549(22.9%) | 3107(30.4%) | 6292(34.0%) |  |  | a,b,c |
|  | 40-43 | 6873(19.4%) | 1287(19.0%) | 2212(21.7%) | 3374(18.2%) |  |  | a,c |
|  | ≥44 | 8922(25.2%) | 2437(36.0%) | 2724(26.7%) | 3761(20.3%) |  |  | a,b,c |
| **Female** |  |  |  |  |  | 917.524 | <0.001 |  |
|  | ≤35 | 12914(25.3%) | 1348(21.3%) | 2749(21.4%) | 8817 (27.7%) |  |  | b,c |
|  | 36-39 | 16053(31.4%) | 1496(23.6%) | 4001(31.1%) | 10556(33.1%) |  |  | a,b,c |
|  | 40-43 | 9792(19.2%) | 1219(19.2%) | 2686(20.9%) | 5887(18.5%) |  |  | a,c |
|  | ≥44 | 12292(24.1%) | 2273(35.9%) | 3414(26.6%) | 6605 (20.7%) |  |  | a,b,c |

a: significant difference between the junior high group and the senior high group; b: significant difference between the junior high group and the university group; c: significant difference between the senior high group and the university group.
